# Compatibility effects with touchless gestures

**DOI:** 10.1007/s00221-023-06549-1

**Published:** 2023-01-31

**Authors:** Markus Janczyk

**Affiliations:** Department of Psychology, University of Bremen, Hochschulring 18, 28359 Bremen, Germany

**Keywords:** Stimulus–response compatibility, Simon effect, Response–effect compatibility, Touchless gestures, Compatibility effects

## Abstract

Human actions are suspect to various compatibility phenomena. For example, responding is faster to the side where a stimulus appears than to the opposite side, referred to as stimulus–response (S–R) compatibility. This is even true, if the response is given to a different stimulus feature, while location itself is irrelevant (Simon compatibility). In addition, responses typically produce perceivable effects on the environment. If they do so in a predictable way, responses are faster if they produce a (e.g., spatially) compatible effect on the same side than on the other side. That it, a left response is produced faster if it results predictably in a left effect than in a right effect. This effect is called response-effect (R–E) compatibility. Finally, compatibility could also exist between stimuli and the effects, which is accordingly called stimulus-effect (S–E) compatibility. Such compatibility phenomena are also relevant for applied purposes, be it in laparoscopic surgery or aviation. The present study investigates Simon and R–E compatibility for touchless gesture interactions. In line with a recent study, no effect of R–E compatibility was observed, yet irrelevant stimulus location yielded a large Simon effect. Touchless gestures thus seem to behave differently with regard to compatibility phenomena than interactions via (other) tools such as levers.

## Compatibility effects with touchless gestures

Many working and leisure environments involve interactions with tools or transformed movements. Such interactions are often suspect to various compatibility phenomena involving stimuli, responses, and the effects following from bodily movements. The present study addresses compatibility phenomena for interactions involving touchless gestures.

### Compatibility phenomena and their applications

Psychological research has uncovered several phenomena of compatibility between stimulation and responses and, as will be introduced below, between responses and their contingent environmental changes, so called action effects.

Arguably the most widely investigated instance is that of stimulus–response (S–R) compatibility (Fitts and Deininger 1954; Fitts and Seeger 1953; see Proctor and Vu 2006, for a comprehensive overview). If stimuli and responses overlap on a certain (e.g., spatial) dimension (“set-level compatibility”; Kornblum et al. 1990), responses are given faster when they are required on the side of the stimuli instead of the opposite side (“element-level compatibility”; Kornblum et al. 1990). One particular example is when stimuli occur on the left versus right side of a computer screen and require a left versus right manual key press response. Responding to a left (right) stimulus with a left (right) key press (S–R compatible) is then faster (and less error-prone) than responding to left (right) stimulus with a right (left) key press (S–R incompatible). Note that in this case, the stimulus location is task-relevant as it defines the correct response.

However, even if stimulus location is task-irrelevant and thus participants are required to respond to a different stimulus feature than to its location, the relation between stimulus and response location affects response times (RTs). Such observations go back to studies by Simon (e.g., 1969). In a typical experiment, stimuli may be colored circles, presented on the left versus right side, and requiring a left versus right response to the color (the task-relevant feature). Although stimulus location is thus task-irrelevant, RTs are shorter if stimulus location and response location are the same (Simon compatible) than when they are not (Simon incompatible; see, e.g., Hommel 2011, for a review).

A third relation of importance to the present study is response–effect (R–E) compatibility. Consider a study where participants produce in one block of trials a left visual effect (e.g., the brief onset of a circle) with a left response and a right visual effect with a right response (R–E compatible). In a different block, this assignment is reversed and a left response produces a right effect and a right response produces a left effect (R–E incompatible). Applying both R–E compatibility conditions in separate blocks allows perfect predictability of the responses’ effects. Even though the effects become visible only when RT has already been measured, RTs are shorter in R–E compatible than in incompatible blocks (Kunde 2001; see also, e.g., Janczyk et al. 2017; Pfister and Kunde 2013). The theoretical idea behind R–E compatibility effects is the ideomotor principle, a philosophical idea dating back to the nineteenth century (Harleß 1861; Herbart 1825; James 1890; see Pfister and Janczyk 2012, and Stock and Stock 2004, for articles on the ideomotor principle’s history). Essentially, the idea is that people first learn to associate bodily movements with their contingent effects. Later then, bodily movements are selected as actions by mentally anticipating their associated effects. Having been criticized in the beginning of the twentieth century (Thorndike 1913), the ideomotor principle was brought back to psychological research by Greenwald (1970). Since then, its basic ideas have received much empirical evidence (e.g., Elsner and Hommel 2001; Janczyk et al. 2022; Kunde 2001, 2003; Paelecke and Kunde 2007; for reviews from different perspectives, see Badets et al. 2016; Hommel et al. 2001; Janczyk and Kunde 2020; Shin et al. 2010). R–E compatibility is typically taken to indicate an anticipatory mental representation of the pursued effect that primes the compatible response (Janczyk and Lerche 2019), much as an exogenous stimulus would do.

Finally, a compatibility relation can also exist between the stimuli and the effects (of the responses given to the stimuli), thus called stimulus-effect (S–E) compatibility. In many instances, and in the present study as well, S–R, R–E, and S–E compatibility cannot be varied orthogonally. Rather, if the relations of S–R and R–E are both compatible or both incompatible, the S–E relation is compatible as well. Otherwise, the S–E relation is incompatible. In this way, S–E compatibility can be seen as an interaction of S–R and R–E compatibility.

Compatibility phenomena like those reviewed so far have also been at the heart of more applied studies (see Proctor and Vu 2016), and of particular interest for the present study is R–E compatibility in these cases.

A first example is the attitude indicator in aviation indicating deviations from level flight. Two variants exist that visualize the deviation in opposite ways: Western airplanes use a “moving horizon” display where the plane remains fixed, but the horizon rotates into the opposite direction of the control movement. In contrast, Russian airplanes use a “moving plane” display where the horizon remains fixed, but the plane rotates into the same direction as the control movement (see Previc and Ercoline 1999, for a review). Originally discussed under the term “control-display compatibility”, the former represents a case of R–E incompatibility, while the latter could be conceived as R–E compatible (see also Ding and Proctor 2017; Müller et al. 2019; Yamaguchi and Proctor 2010). Indeed, when focusing on RTs as the dependent variable, a typical R–E compatibility effect was observed (Janczyk et al. 2015).

Another example involves tool use, and in particular first-class levers that invert the effector movement via their pivot point. Thus, if the hand moves into one direction, the tip of the tool moves into the opposite direction. Again, this can be conceived as an R–E incompatible relation. Laparoscopic surgery is an example of practical relevance where the inversion has been termed the “fulcrum effect” (see also Heuer and Sülzenbrück 2009; Sülzenbrück and Heuer 2009), coming with disadvantages when compared to classical surgery (Savader et al. 1997; see also Heemskerk et al. 2006). Kunde et al. (2007) used a custom-built device that was (virtually) connected to visualized lever movements on a computer screen in two different ways, one reverting the hand movement (R–E incompatible) and one where hand and tool movement were into the same direction (R–E compatible). In two experiments, an R–E compatibility effect was observed, which was, additionally, independent of an S–R compatibility effect in Experiment 1 and of a Simon effect in Experiment 2 (see also Kunde et al. 2012; Janczyk et al. 2012). These additional effects were coded with regard to the tip of the lever, thus they can be construed as an instance of S–E compatibility.

Further, R–E compatibility plays a role in human–computer interaction when the relation between scrolling direction and display movement is considered. In principle, moving a slider or finger upwards can result in the display moving upward as well (an R–E compatible relation) or moving downward instead (an R–E incompatible relation). For instance, when using a touchscreen on a smartphone, R–E relations are typically compatible (e.g., swiping upward leads to the document moving upward as well). Instead, when using the slider in a document viewer, R–E relations can be incompatible (e.g., moving the slider upward leads to the document moving downward). Empirically, for key presses as responses, a performance difference between both R–E compatibility conditions has been reported in two studies, however, with opposite results. More precisely, performance was better in the R–E incompatible condition in a study by Bury et al. (1982), but better in the R–E compatible condition in a study by Chen and Proctor (2013). This difference may be attributed to the first study having been conducted in a time, where most humans were not used to touch screens and the like.

By and large then, compatibility phenomena, including R–E compatibility, appear to be a rather stable phenomenon in both basic science investigations and in more applied contexts as well.

### Touchless interaction

In all studies mentioned in the previous section, the fingers or hands used as the effectors actually touched the response device during responding. This is true when pressing a response button with a finger, when moving a lever with the hand, or when moving a joystick or steering wheel with one or both hands. Touchless interfaces, where gestures are performed without direct contact to a response device, allow for way more flexibility though (O’Hara et al. 2013) and have been introduced in various contexts recently. One example are hospitals where touchless interaction reduces the necessity to sterilize user interfaces (see, e.g., Cronin and Doherty 2019). Another example are infotainment systems in cars that can be controlled with touchless gestures (Ashley 2014; Zöller et al. 2018) and potentially reduce the amount of visual attention otherwise required to press the correct button (Kim and Song 2014; see also Stecher et al. 2018).

**Fig. 1:**
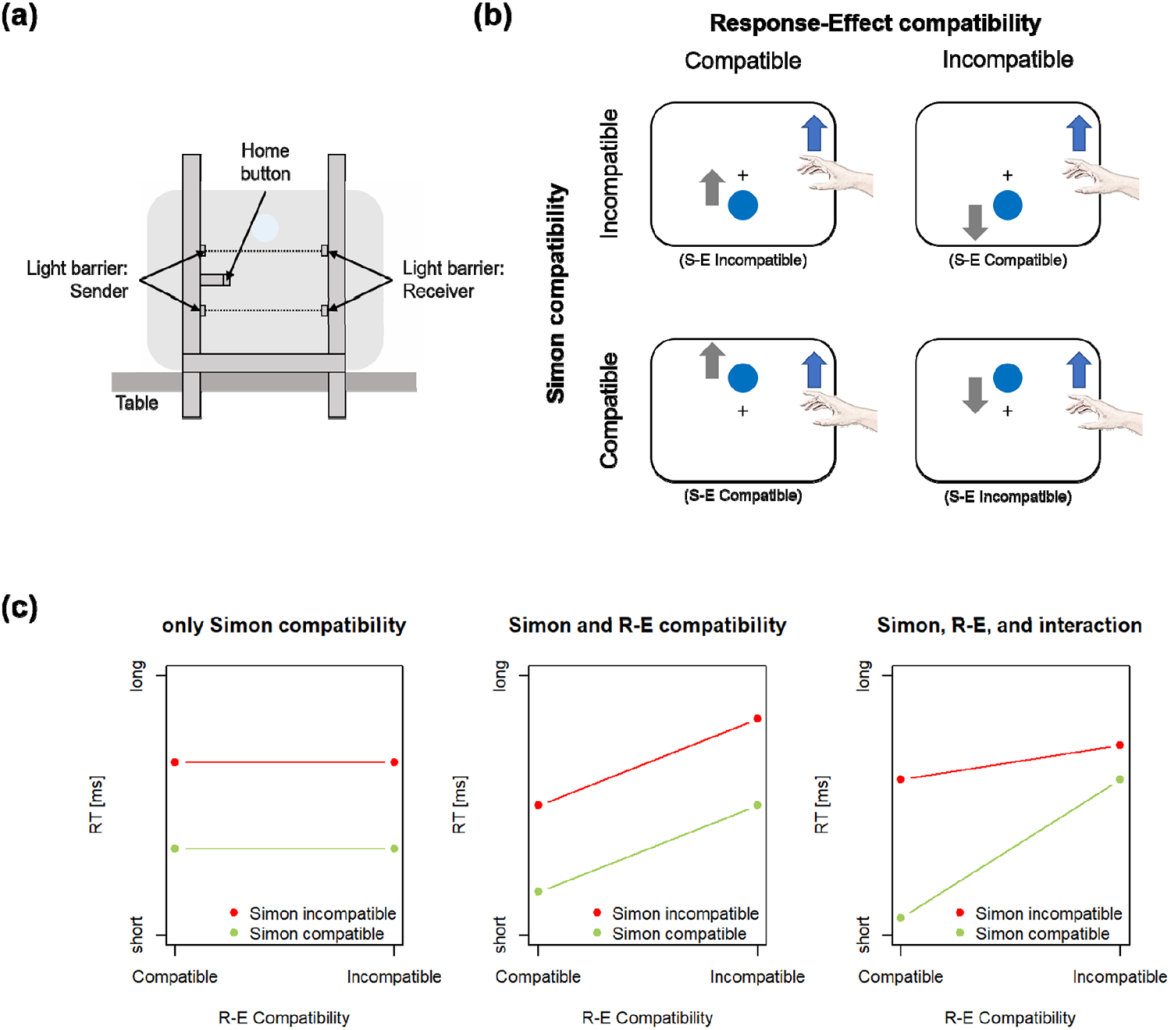
**a** Illustration of the response device used in the present study and in Janczyk et al. (2019). Participants touched a (touch-sensitive) home button with the left side of their right index finger and initiated their movement from this position. An up- or downward gesture response was detected via light barriers. **b** Illustration of the stimulus setup and the resulting compatibility conditions. The blue circle is an example for a stimulus requiring an upward gesture (indicated by the blue arrow). The movement direction of the stimulus, that is, the effect, is indicated with the gray arrow. Gesture and effect movement are into the same direction in response-effect (R–E) compatible conditions and in opposite directions in R–E incompatible conditions. **c** Predicted response time (RT) patterns when different types of compatibility are effective for touchless gestures (see Sect. "The present study" for more details). The interaction of Simon and R–E compatibility can be construed as an effect of stimulus-effect (S–E) compatibility

Janczyk et al. (2019) investigated compatibility effects, with a focus on R–E compatibility, in a context borrowed from interacting with an infotainment system in a car. Responses were touchless up- or downward gestures collected with the response device illustrated in Fig. 1a, which was also used in the present study. In most experiments, participants saw three vertically arranged icons on the screen. The middle item was surrounded by a frame and thus in the ‘focus’. R–E compatibility was defined with respect to the direction of the continuous scrolling movement of the three items. Hence, in the R–E compatible condition, an upward gesture made the items move upward as well, while the items moved downward in the R–E incompatible condition. Note, however, that there exists a further yet subtle compatibility relation: in the R–E compatible condition, an upward movement also resulted in the lower item being brought into the focus. Similarly, an upward movement in the R–E incompatible condition would result in the upper item being in the focus. Thus, if bringing an item into focus was conceived as the actor’s goal, one could also speculate whether the R–E incompatible condition would be superior as here goal location and gesture direction were compatible.

Somewhat unexpected though, Janczyk et al. (2019) did not observe stable signs of an R–E compatibility effect across several experiments. Rather (see also Footnote 1 for more details in the context of the present study’s results), the R–E compatibility effect was not always significant in the expected direction and once significant but reversed, that is responses were faster in R–E incompatible trials. Yet, touchless gestures were not immune to compatibility phenomena per se, as an S–R compatibility effect was still observed (even though S–R compatibility was not manipulated independently of R–E compatibility; their Exp. 3).

### The present study

The results of Janczyk et al. (2019) deviate from numerous other studies with different responses and stable R–E compatibility effects. As interesting as this is, it appears necessary to conceptually replicate these results to put it on firmer ground. This is the main purpose of the present study and two experiments will be presented here that follow-up the study by Janczyk et al. in a simplified setting. The same response device was used (see Fig. 1a). The stimulus, however, was a yellow or blue circle occurring above or below the screen center and participants were required to make an up- or downward gesture according to the stimulus color. The gesture made the circle move up- or downward in a continuous way. In the R–E compatible condition, it moved into the direction of the gesture, and it moved into the opposite direction in the R–E incompatible condition. In addition, stimulus location and gesture direction created conditions of Simon compatibility: if the stimulus was above (below) and required an upward (downward) gesture, this would be Simon compatible, and otherwise Simon incompatible. In contrast to the S–R compatibility conditions in Janczyk et al., the stimulus location in the present study is thus task-irrelevant. The four design cells resulting from crossing R–E and Simon compatibility are visualized in Fig. 1b. Should the results replicate the previous study, it is of theoretical relevance to determine the difference between touchless gestures and other responses used in related studies.

Three different result patterns can be predicted under different scenarios of existing compatibility effects (see Fig. 1c). All scenarios assume that a Simon compatibility effect exists, although Janczyk et al. (2019) only considered S–R compatibility, that is, the case where stimulus location is task-relevant. (1) If, for touchless gestures, R–E compatibility does not play a role, only a main effect of Simon compatibility would emerge with shorter RTs in Simon compatible trials than in Simon incompatible trials (see left panel of Fig. 1c). This would be the likely result against the background of the Janczyk et al. study. (2) If, however, the non-existence of an R–E compatibility effect in the Janczyk et al. study was due to a peculiarity of the visual display and/or the reversed compatibility relation when considering the goal of an actor, one may assume an effect of R–E compatibility to be present with the present setup. In the simplest case, the two main effects of R–E and Simon compatibility would emerge with faster responses in the respective compatible compared with the incompatible conditions (see middle panel of Fig. 1c). (3) If the effects play a role for touchless gestures, it is conceivable that the relation between stimuli and the effects, that is, S–E compatibility, affects responses as well (see Kunde et al. 2007, with lever movements). As mentioned above, S–E compatibility results from jointly considering S–R and R–E relations and their interaction. More precisely, if a trial is both R–E and Simon compatible or incompatible, the S–E relation would also be compatible. Thus, responses in these cells would perhaps become (even) faster. In contrast, if the trial is either R–E *or* Simon compatible, with the other relation being incompatible, it would be also S–E incompatible. The respective responses in these cells would thus become slower. This pattern would result in a statistical interaction of R–E and Simon compatibility as is visualized in the right panel of Fig. 1c.

## Experiment 1

### Methods

#### Participants

Twenty-four people (mean age = 24 years, 16 female) from the Tübingen (Germany) area participated for course credit or monetary compensation. Participants were naïve regarding the hypotheses of this experiment, reported normal or corrected-to-normal vision, and signed informed consent prior to data collection. The smallest effect size for an effect representing S–R compatibility in Janczyk et al. (2019) was *η*_p_^2^ = 0.32. The power to detect an effect of this size (or larger) is 1 – *β* = 0.88 with *n* = 24 participants.

#### Stimuli and material

The experiment was run on a standard PC connected to a 17 in. CRT monitor in a dimly lit, sound attenuated experimental cabin. Stimuli were a yellow and blue circle presented against a black background. The response device was the same as used by Janczyk et al. (2019; see Fig. 1a). Participants needed to touch a touch-sensitive home button with a small part of the right index-finger. Two light barriers 4 cm above and below the home button allowed to register the direction of the gesture, as crossing a light barrier closed a switch, which was registered by the experimental program.

#### Task and procedure

The participants’ task was to make an up- or downward gesture with the right hand in response to the stimulus color. Participants sat on a chair in front of the apparatus in a comfortable manner. A trial began when they touched the home button with their right index-finger. In this posture, the right arm was elevated but bended. The general posture was the same for all participants, although it differed slightly depending on, for example, the participants’ height. When a participant touched the home button, a small fixation cross was presented (250 ms). After a blank screen (250 ms), the stimulus appeared either below or above the screen center, half-way between the center and the upper or lower end of the visible screen. Participants had a maximum of 2500 ms to initiate the gesture movement, and RT was measured from stimulus onset until the home button was not touched any longer. When the correct gesture was executed, the stimulus circle started a continuous up- or downward movement until it left the visible screen. The direction of the movement depended on the current R–E compatibility condition: in R–E compatible blocks, it was into the same direction as the gesture, and in R–E incompatible blocks, it was into the opposite direction. Errors (missing response, wrong gesture direction) were fed back in written form to the participant for 1000 ms. The next trial started after an inter-trial interval of 1000 ms.

One block of trials comprised 52 trials, resulting from 13 repetitions of 2 stimulus colors (yellow vs. blue) × 2 stimulus locations (above vs. below screen center), presented in random order. Participants began with two short familiarization blocks with 10 randomly drawn trials, one for each R–E compatibility condition. These were followed by 10 full blocks, with 5 consecutive ones for each R–E compatibility condition. The order of R–E compatibility conditions as well as the S–R mapping were counterbalanced across participants.

#### Design and analyses

Trials were classified according to their R–E compatibility (compatible: effect movement into the direction of gesture; incompatible: effect movement into the direction opposite to the gesture) and Simon compatibility (compatible: stimulus location and direction of the gesture were the same; incompatible: stimulus location and direction of the gesture were different).

For all analyses, the familiarization blocks, the first blocks of each R–E compatibility condition, trials without responses, and trials in which the time from leaving the home button until crossing one of the light barriers exceeded 100 ms (to ensure that participants did not leave the home button and then decide on the direction in a trial) were excluded. Additionally, for RT analyses, erroneous trials and trials with an RT deviating more than 2.5 *SD*s from the individual cell mean were excluded.

Mean correct RTs and percentages error (PE) were submitted to 2 × 2 ANOVAs with R–E compatibility (compatible vs. incompatible) and Simon compatibility (compatible vs. incompatible) as repeated measures. Effect sizes are reported as partial *η*^2^ (*η*_p_^2^) and *α* = 0.05 was adopted as the significance level.

All data from this study are publicly available at https://osf.io/4sgea/. The experiments reported here were not preregistered.

### Results

**Fig. 2:**
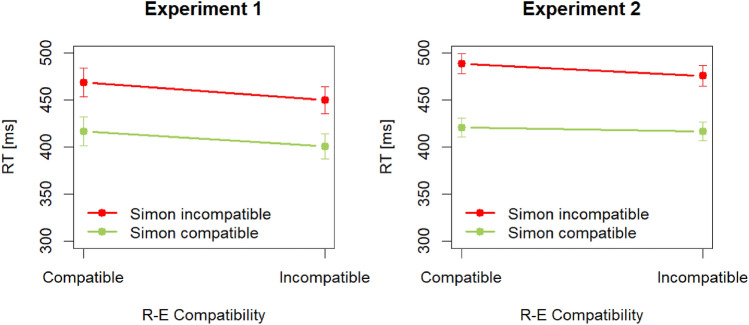
Mean correct response times (RTs) in milliseconds (ms) as a function of Simon and response-effect (R–E) compatibility separately for Experiment 1 (left panel) and Experiment 2 (right panel). Error bars are (between-subject) standard errors of the means

Mean correct RTs (2.91% of the trials were excluded as outliers) are visualized in Fig. 2 (left panel) and are summarized in Table 1. Overall, they were shorter in incompatible (426 ms) compared with compatible (443 ms) R–E conditions, *F*(1,23) = 7.99, *p* = 0.010, *η*_p_^2^ = 0.26. In addition, they were longer in incompatible (460 ms) than in compatible (409 ms) Simon conditions, *F*(1,23) = 142.96, *p* < 0.001, *η*_p_^2^ = 0.86. The interaction was not significant, *F*(1,23) = 0.38, *p* = 0.546, *η*_p_^2^ = 0.02.

**Table 1:** Mean correct response times in milliseconds/percentages errors as a function of Simon and response-effect (R–E) compatibility

	Experiment 1		Experiment 2	
	Simon compatibility		Simon compatibility	
R–E compatibility	Compatible	Incompatible	Compatible	Incompatible
Incompatible	401/1.43	450/4.05	417/1.28	476/3.87
Compatible	417/1.19	469/4.43	421/1.04	489/4.34

PEs are provided in Table 1. More errors were committed in incompatible than in compatible Simon conditions, *F*(1,23) = 16.13, *p* = 0.001, η_p_^2^ = 0.41. Neither the main effect of R–E compatibility, *F*(1,23) = 0.01, *p* = 0.905, *η*_p_^2^ < 0.01, nor the interaction were significant, *F*(1,23) = 1.12, *p* = 0.302, *η*_p_^2^ = 0.05.

#### Exploratory analysis

**Fig. 3:**
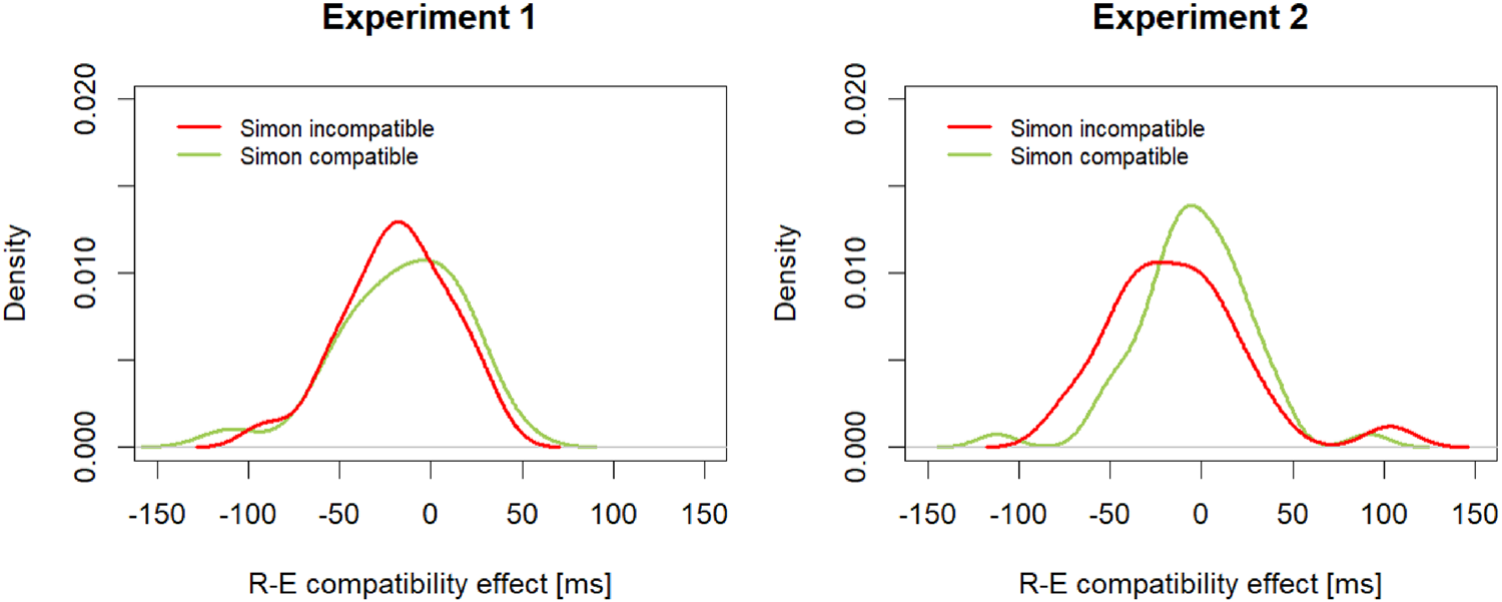
Kernel density estimations of the response-effect (R–E) compatibility effect (= incompatible – compatible R–E conditions) in milliseconds (ms) as a function of Simon compatibility, separately for Experiment 1 (left panel) and Experiment 2 (right panel)

An on-average small or zero effect of R–E compatibility can also result from some participants showing a positive and some participants showing a negative R–E compatibility effect. In this case, the distribution of R–E compatibility effects would be expected bimodal. As can be seen in Fig. 3 (left panel), the distribution is unimodal with a center close to zero.

### Discussion

This experiment was a first one to distinguish between the three predicted result patterns illustrated in Fig. 1c. The results clearly are not in line with the middle and right panel of that figure. Thus, they point to Simon compatibility being the only effective compatibility relation. Notably, R–E compatibility had an effect, yet RTs were (slightly, but significantly so) shorter in R–E incompatible than in R–E compatible trials. Despite this aspect, the data are most in line with the prediction visualized in the left panel of Fig. 1c. Before drawing strong conclusions, Experiment 2 will be reported, which is a close replication of Experiment 1 with two changes: The movement distances of the stimulus circles were equated for all conditions, and a larger sample of participants was tested. The larger sample size allows more precise estimation and increases statistical power. In particular, it is important to assess whether the reversed R–E compatibility effect would replicate under these conditions.

## Experiment 2

### Methods

#### Participants

Forty-eight people (mean age = 24.35 years, 41 female) from the Tübingen (Germany) area participated for course credit or monetary compensation. With the same considerations as given for Experiment 1, the power increases to 1 – *β* > 0.99 with *n* = 48 participants.

#### Stimuli, material, task, procedure, design, and analyses

This experiment was very similar to Experiment 1 with one change made to the effect movement. More precisely, when the effect moved toward the screen center, its movement stopped there (instead of continuing until it was outside the visible screen area, as in Experiment 1). With this change, all effect movements were of the same length and duration.

### Results

Mean correct RTs (2.73% of the trials were excluded as outliers) are visualized in Fig. 2 (right panel) and are summarized in Table 1. Overall, they were (descriptively) shorter in incompatible (446 ms) compared with compatible (455 ms) R–E conditions, although the main effect did not reach significance, *F*(1,47) = 3.58, *p* = 0.065, *η*_p_^2^ = 0.07. In addition, they were longer in incompatible (483 ms) than in compatible (419 ms) Simon conditions, *F*(1,47) = 484.43, *p* < 0.001, *η*_p_^2^ = 0.91. The interaction was also significant, *F*(1,47) = 4.36, *p* = 0.042, *η*_p_^2^ = 0.08, reflecting a larger effect of R–E compatibility in Simon incompatible than in Simon compatible conditions.

PEs are provided in Table 1. More errors were committed in incompatible than in compatible Simon conditions, *F*(1,47) = 55.93, *p* < 0.001, *η*_p_^2^ = 0.54. Neither the main effect of R–E compatibility, *F*(1,47) = 0.21, *p* = 0.649, *η*_p_^2^ < 0.01, nor the interaction were significant, *F*(1,47) = 1.78, *p* = 0.188, *η*_p_^2^ = 0.04.

#### Exploratory analysis

As can be seen in Fig. 3 (right panel), the distribution of R–E compatibility effects is unimodal with a center close to zero.

### Discussion

The clearest effect in this experiment was again that of Simon compatibility with faster responses in Simon compatible than in Simon incompatible trials. RTs were also shorter in R–E incompatible trials compared with R–E compatible trials, although in this experiment, the main effect was not significant. In addition, the interaction was significant with the R–E compatibility difference being larger in Simon incompatible trials. Yet, this pattern is not in line with the predicted interaction visualized in the right panel of Fig. 1c.

## General discussion

The present study investigated compatibility phenomena with touchless gestures as responses. Allowing for more flexibility as compared with traditional interaction devices (O’Hara et al. 2013), they can, for example, be used to interact with infotainment systems in cars (Ashley 2014).

Compatibility phenomena such as S–R compatibility (Fitts and Deininger 1954; Fitts and Seeger 1953), Simon compatibility (Simon 1969), and R–E compatibility (Kunde 2001) have been investigated and demonstrated in several applied contexts in previous research (e.g., Bury et al. 1982; Chen and Proctor 2013; Janczyk et al. 2012, 2015; Kunde et al. 2007, 2012; Müsseler and Skottke 2011; Müller et al. 2019, 2021; Yamaguchi and Proctor 2010).

Janczyk et al. (2019) investigated compatibility effects for touchless gestures (in an automobile context). Across several experiments, these authors did not obtain evidence for an effect of R–E compatibility though. Yet, touchless gestures are not immune to compatibility effects, as a strong S–R compatibility effect was obtained in that study.

The present study reports two experiments that further addressed compatibility phenomena for touchless gestures. In contrast to the just mentioned study by Janczyk et al. (2019), the automobile context was removed to investigate whether the absent R–E compatibility effect was due to some peculiarities of the particular setup. In addition, in the previous study, S–R compatibility was investigated, that is, stimulus location was task-relevant. In the present experiments, I investigated Simon compatibility effects, where stimulus location is task-irrelevant.

The results regarding Simon compatibility are straightforward: even though stimulus location was task irrelevant, Simon compatibility had a large effect on RTs in both experiments, with faster responses in compatible than in incompatible trials. Thus, both S–R and Simon compatibility clearly seem to affect the initiation of touchless gestures. Regarding R–E compatibility, the results reported above are slightly ambiguous. In both experiments, RTs were shorter in incompatible than in compatible trials. In Experiment 1, this effect was even significant; in Experiment 2, with a larger sample size, the effect was not significant. To resolve this situation, I computed Bayes Factors BF_10_ for the main effect of R–E compatibility in both experiments using the BayesFactor package in R (Morey and Rouder 2018, function anovaBF() with standard settings and 100,000 iterations). These analyses yielded slightly more evidence for the presence of an effect in Experiment 1, BF_10_ = 2.12 (± 0.78%), and more evidence for the absence of an effect in Experiment 2, BF_10_ = 0.38 (± 1.03%). In addition, the small (reversed) or zero effect of R–E compatibility on average is not due to a bimodal distribution of individual R–E compatibility effects (see Fig. 3). Hence, at present the most warranted conclusion seems that R–E compatibility does not affect the initiation of touchless gestures^1^. In sum then, the results most closely match the predictions illustrated in the leftmost panel of Fig. 1c.

However, given the descriptive pattern in both experiments, let us assume for a moment that the reversed R–E compatibility effect exists. Then it is appropriate to ask for the cause of this reversal. While there appears to be no reason within the design, I can nonetheless offer a post-hoc speculation. Remember that R–E compatibility proper is defined as the compatibility between the direction of the gesture and of the effect. Now, in the present setup (and in more naturalistic setups likely as well), after making an upward (downward) gesture, participants make a downward (upward) movement to return the hand to its initial position, which, in the present context, is the home button. This return-movement, in turn, is then compatible to the effect movement direction in R–E incompatible conditions and vice versa and could indeed be the cause of the reversed R–E compatibility effect. Of course, this requires the assumption that this “second” part of the response was co-represented, too, to affect RTs. Note, however, that the initial gesture direction must have been coded as well, as otherwise the Simon effect should be reversed, too. Albeit being a post-hoc speculation, such an explanation certainly deserves future investigation to determine the relevant aspect of a gesture in creating compatibility effects.

Irrespective of the just offered speculation, the cautious interpretation of the present experiments and that reported by Janczyk et al. (2019; see also Footnote 1) is that no R–E compatibility effect exists in the sense that the initiation of the gesture is not affected by its contingent effect. This outcome is particularly interesting considering that for many tools a predominance of action effects has been reported (see Sutter et al. 2013, for a review). As one example, Müsseler and Skottke (2011) used a lever device that allowed to orthogonally cross S–R, R–E, and S–E compatibility. This study replicated earlier studies in showing that an inverted lever movement (i.e., R–E incompatibility) causes costs, but additionally demonstrated that “this effect overrules all other relationships” (p. 388). Thus, for levers it appears as if stimulus-based compatibility relations are of less importance compared with effect-based compatibility relations. In contrast, for touchless gestures, as used in the present study, the opposite seems the case. As a practical consequence, users could be encouraged to choose their preferred R–E compatibility relation to further improve user experience (in case reliable individual differences exist).

One aspect of the design warrants further discussion. It is conceivable that downward gestures are carried out faster than upward gestures due to gravity. In the present study, however, RTs index the time until a decision for a down- or upward gesture is made, and it appears reasonable that this decision per se is not affected by gravity. Indeed, in a post-hoc analysis, RTs did not differ significantly between both movement directions in both experiments. And even if there were a difference, both R–E and Simon compatible and incompatible trials were combined with down- and upward movements, and thus movement direction was not confounded with any relevant independent variable.

The theoretically interesting aspect of the present results is: why are touchless gestures special with respect to their susceptibility to effect-based compatibility relations? There is one obvious difference between the present study (and that by Janczyk et al. 2019) and those reviewed in the introduction that revealed effect-based compatibility effects: in the latter studies, participants responded with response devices (response keys, joysticks, levers, steering wheels) that required direct contact with the (manual) effectors. Thus, participants experienced tactile feedback from touching and operating with the response device the movement of which brought about the sensory changes, that is, the “environment-related effects”. With touchless gestures, in contrast, participants do not directly touch a response device and they do not receive this tactile feedback (while proprioceptive feedback still exists). Ideomotor theory allows for the anticipation of different types of effects, including proprioceptive and tactile ones, as a means of response selection (“body-related effects”; see Pfister 2019). Unfortunately, the role of such action effects has rarely been addressed in empirical research, though some approximations of manipulating tactile effects have been published and revealed R–E compatibility effects (e.g., Pfister et al. 2014; Thébault et al. 2018; Wirth et al. 2016). Given that, in typical setups, the critical features (e.g., spatial location) of proprioceptive and tactile effects (and often even the motor efference signal) are confounded (e.g., they are all on the left side), the present results might be taken to suggest that the compatibility typically conceived as one between responses and effects (hence R–E) is actually one between tactile and environment-related effects, thus E–E compatibility 2. Without tactile effects, as in the present case of touchless gestures, no compatibility then exists. Interpreted in this way, the present results help scrutinizing the (functional) roles of the multiple effects that follow a bodily movement and thereby allow more precise formulations of the ideomotor principle (see Pfister 2019, for a broader discussion). Alternatively, Sutter et al. (2013) suggested that in cases with extremely discrepant body- and environment-related feedback, the latter might lose its impact relative to the former. Whether this might be an explanation in the present case is unclear; it might as well be that the (relative) lack of tactile feedback creates difficulties in incorporating the environment-related effects.

In sum, the present study replicates results of Janczyk et al. (2019) that touchless gestures may not be suspect to effect-related compatibility phenomena. At the same time, though, they are heavily suspect to stimulus-related compatibility phenomena, even if location is irrelevant (as in Simon compatibility). Future research should aim to determine the relevant feature responsible for *why* touchless gestures are so different from other tools and tool transformations.

## Data Availability

All data from this study are publicly available at https://osf.io/4sgea/.

## Footnotes

1 This conclusion would also be in line with that of Janczyk et al. (2019). In that study, a non-significant trend for a normal R–E compatibility effect was observed for free-choice trials in Experiment 1 and a small, but significant R–E compatibility effect was present in Experiment 2b (only free-choice trials). For the forced-choice trials in Experiments 3a and 3b, the effect of R–E compatibility was reversed; however, this translates directly into a normal S–R compatibility effect. Finally, in the free-choice trials in Experiment 3b, a significant reversed R–E compatibility effect was observed without any obvious S–R relation (because the stimuli occurred in the screen center rather than at the top or bottom of the screen).

2 A related suggestion was made by Pfister and Kunde (2013) who stated that the “the term ‘response’-effect compatibility is perhaps better described as effect–effect compatibility, that is, compatibility between the various re-afferences of a given motor output.” (pp. 647–648).
